# Long Non-coding RNA ASNR Targeting miR-519e-5p Promotes Gastric Cancer Development by Regulating FGFR2

**DOI:** 10.3389/fcell.2021.679176

**Published:** 2021-07-09

**Authors:** Zihao Chen, Yong Li, Bibo Tan, Fang Li, Qun Zhao, Liqiao Fan, Zhidong Zhang, Xuefeng Zhao, Yu Liu, Dong Wang

**Affiliations:** ^1^Graduate School of Hebei Medical University, Shijiazhuang, China; ^2^The Third Department of Surgery, The Fourth Hospital of Hebei Medical University, Shijiazhuang, China; ^3^Department of Pathology, The Fourth Hospital of Hebei Medical University, Shijiazhuang, China

**Keywords:** gastric cancer, long non-coding RNA, Lnc_ASNR, proliferation, invasion, migration

## Abstract

Gastric cancer (GC), as a common gastrointestinal tumor, is an important cause of death from cancer all around the world. Long non-coding RNAs (lncRNAs), a novel class of transcripts, have attracted great attention of researchers. However, the mechanisms of the clinical significance of most lncRNAs in human cancer are mainly undocumented. This research desires to explore the clinical significance, biological function, and mechanism of Lnc_ASNR (apoptosis suppressing-non-coding RNA) in GC. Cell proliferation, cell cycle, cell migration, and invasion abilities were respectively determined by 3-(4,5)-dimethylthiahiazo (-z-y1)-3,5-di-phenytetrazoliumromide (MTT), flow cytometry, wound healing, and Transwell assay (Sigma-Aldrich, St. Louis, MO, United States). The association of Lnc_ASNR, miR-519e-5p, and fibroblast growth factor receptor 2 (FGFR2) was evaluated *via* luciferase reporter experiments. The tumor xenograft assay was conducted to confirm the results of cell experiments. High expressed Lnc_ASNR was detected in both GC cells and tissues using qRT-PCR. Downregulated Lnc_ASNR could reduce proliferation, migration, and invasion in GC cells, while upregulated Lnc_ASNR could promote the cell proliferation, migration, and invasion. Moreover, the effect of Lnc_ASNR on migration and invasion ability is closely related to epithelial-mesenchymal transition (EMT). The bioinformatics analysis, luciferase assay, and Western blot demonstrated that Lnc_ASNR inhibited miR-519e-5p expression but increased FGFR2 expression. Lnc_ASNR and FGFR2 were both targeted to miR-519e-5p, and they were negatively correlated with the expression of miR-519e-5p. All investigations indicated that Lnc_ASNR functioned as a ceRNA targeting miR-519e-5p and facilitated GC development by regulating the pathway of miR-519e-5p/FGFR2.

## Introduction

Gastric cancer (GC) is a prevalent malignant tumor around the world with the sixth incidence rate and second mortality rate, which seriously affects human health (Bray et al., 2018). The advanced diagnosis and therapy of GC has achieved development in recent years. However, many limitations existed that hinder the effectiveness of GC treatment. The early symptoms of most GC patients are not obvious, and diagnosis delay becomes the main obstacle of GC treatment and prognosis. The combination of gastroscopy and biopsy is the universally recognized gold standard for GC diagnosis (Virgilio et al., 2017; den Hollander et al., 2019). However, its application in GC screening is still limited because it is not easy to be accepted by patients due to the invasiveness and high cost. At present, the most commonly used tumor biomarkers in clinical are insufficient to completely replace biopsy, so it is of great significance to explore more specific and sensitive biomarkers for early diagnosis of GC to improve the clinical treatment effect.

Long non-coding RNA (lncRNA) is an RNA molecule with a length between 200 and 100,000 nucleotides (nt), which can be transcribed independently. lncRNA is molecularly similar to mRNA, but there is no identifiable potential to encode functional proteins (Ulitsky, 2016). Researchers confirmed that lncRNA contains a significant biological function among several diseases (Montes et al., 2021; Zhang F. et al., 2021; Zhang Y. et al., 2021; Zhu et al., 2021). It is related to tumor development, chemotherapy sensitivity, and prognosis and especially plays a vital role in the resistance of multiple tumor chemotherapy drugs, especially in GC (Zhang and Wang, 2021), prostate cancer (Shang et al., 2019), and pancreatic cancer (Zhou et al., 2020). The expression of lncRNA is abnormal in a wide range of cancers. lncRNA demonstrates an essential influence in facilitating and inhibiting cancer occurrence and development, which proves its clinical potential as a biomarker and therapeutic target (Kim et al., 2018; Ramnarine et al., 2019; Wang Y. L. et al., 2019; Olivero et al., 2020). Recent findings indicated that lncRNA can interact with proteins, RNA, and lipids, thereby producing cancer signal transduction (Lin and Yang, 2018). In addition, lncRNAs, as competing endogenous RNAs (ceRNAs) or microRNA sponge, can regulate expression by competitively binding to microRNA (Song et al., 2017; Sun et al., 2017; Huang et al., 2019). Researchers have found that most lncRNAs are upregulated or downregulated expressly in tissues and serum plasma of patients with GC (Zhang et al., 2018; Wang C. J. et al., 2019; Guo et al., 2020; Yuan et al., 2020). Therefore, lncRNA can be used as a potential tumor marker for GC diagnosis.

Lnc_ASNR (non-coding RNA that inhibits apoptosis) is a nuclear-reserved lncRNA. Lnc_ASNR located in the chr14-q22.2 region is a newly discovered lncRNA associated with cancer and other diseases. Compared with normal tissues, Lnc_ASNR expression is significantly increased in four tumor tissues. Chen’s group (Chen et al., 2016) has found that Lnc_ASNR promotes the reduction of cytoplasmic AUF1 levels and inhibits AUF1-mediated degradation of Bcl-2 mRNA in colon cancer RKO cells, which leads to high expression of Bcl-2 and significantly inhibits cell apoptosis. However, the role and mechanism of Lnc_ASNR in tumorigenesis and development are unclear, which suggested that the study of the impact of Lnc_ASNR on tumors remains to be explored. Herein, we first evaluated whether Lnc_ASNR changes in the expression of lncRNA in GC and further clarified the biological function of Lnc_ASNR and the molecular mechanism that regulates the occurrence and development of GC. The research results help to identify important molecules that effectively regulate the occurrence and development of GC, which will provide a theoretical basis for clinical molecular diagnosis of GC and the development of targeted drugs. Our results exhibit important scientific significance and potential clinical application value for improving patient prognosis and enhancing survival rate.

## Materials and Methods

### Clinical and Histologic Evaluation of Human Tissues

In this study, 76 paired tumor tissues and adjacent non-tumor tissues were collected from GC patients at the Fourth Hospital of Hebei Medical University from 2017 to 2020. The GC diagnosis was histopathologically confirmed. Clinical pathological features, including gender, age, tumor-node-metastasis (TNM) staging, and tumor size, were summarized. None of them received preoperative treatment, including chemotherapy or radiotherapy. After obtaining the tissues, the samples were immediately placed in liquid nitrogen and then stored at −80°C. The research was approved by the Research Ethics Committee at the Fourth Hospital of Hebei Medical University (Shijiazhuang, China). All patients were informed and gave their consent.

### Cell Culture

Four GC cell lines (MKN45, MKN28, AGS, and HGC27) and gastric mucosal epithelial cell (GES-1) were purchased from the Cell Bank of the Chinese Academy of Sciences (Shanghai, China). All cells were cultured in Roswell Park Memorial Institute (RPMI) 1640 medium (GIBCO, Thermo Fisher Scientific, Waltham, MA, United States) containing 10% fetal bovine serum (FBS) (BI), 100 U/ml penicillin, and 100 mg/ml streptomycin (Invitrogen, Thermo Fisher Scientific) at 37°C with 5% CO_2_.

### RNA Extraction and Quantitative Real-Time PCR

RNAiso Plus (TaKaRa, Tokyo, Japan) was utilized to extract total RNA from cells and tissue samples according to manufacturer’s information. The OD260/OD280 ratio ranging from 1.9 to 2.1 tested by UV spectrophotometer is qualified. Reverse transcription polymerase chain reaction was performed by LnRctue lncRNA First-Strand cDNA kit (TIANGEN, Beijing, China) and LnRctue lncRNA qPCR kit (SYBR Green) (TIANGEN) for quantitative real-time PCR (qRT-PCR) according to manufacturer’s information. ABI 7500 real-time PCR system (Applied Biosystems, Waltham, MA, United States) was utilized to conduct qRT-PCR and collect data. GAPDH was used as a loading control. Our qRT-PCR results were analyzed and expressed relative to threshold cycle (C_*t*_) values, and then converted to fold changes. The specific primer sequences used in our study are provided in the Supplementary Table 1.

### Vector Construction and Transfection and siRNA Transfection

Based on manufacturer’s information, Lipofectamine 2000 (Invitrogen) was used to transfect plasmid vectors and siRNAs into GC cells. The plasmid vector pcDNA3.1 used for Lnc_ASNR overexpression in MKN28, the siRNA (three individual ASNR siRNAs) used for Lnc_ASNR knockdown in MKN45 cells, miR-519e-5p inhibitor, and miR-519e-5p mimics, and the negative control (vector or NC) were obtained from Invitrogen. To upregulate ASNR, the coding sequence was increased based on the manufacturer’s instructions and subcloned into the pcDNA3.1 (+) vector (Invitrogen). Then Lipofectamine 2000 (Invitrogen) was used to transfect MKN28 cells with a negative control vector or a ASNR-expressed plasmid. In order to downregulate ASNR, Lipofectamine 2000 (Invitrogen) was utilized to transfect MKN28 cells with the target sequence of negative control siRNA. After being transfected for 48 h, cells were obtained for qRT-PCR and Western blot experiments. The siRNA sequences used in our study are provided in the Supplementary Table 2.

### Lentiviral Infection

The MKN45 cell suspension was seeded onto a six-well plate at concentration of 3 × 10^4^ cells/well, and the cells were grown to a density of 20%. We have created two different groups, sh-ASNR transfected with shASNR green fluorescent protein (GFP) lentivirus and shNC (GENECHEM) transfected with empty GFP lentivirus, respectively. An appropriate amount of lentivirus was added according to the differences in infection (MOI = 20). After 3 days of transfection, a fluorescence microscope was used to observe GFP-tagged gene expression. Cells with transfection efficiency > 80% were selected for further analyses.

### Cell Proliferation Assays

The cell viability of MKN45 cells transfected with si-ASNR and MKN28 cells transfected with pcDNA3.1-ASNR was evaluated *via* MTT Cell Proliferation and Cytotoxicity Assay Kit (Solarbio, Beijing, China) based on the manufacturer’s guidance. Transfected MKN-45 cells and MKN28 cells (5 × 10^4^/well) were incubated with MTT (20 μl/well) at 37°C for 4 h. Next, DMSO was added and mixed for 10 min to ensure the crystals were dissolved. The optical density value was measured at the absorbance 570 nm under Thermo Scientific Microplate Reader Multiskan MK3 (Thermo Fisher Scientific).

### Flow Cytometry for Cell Cycle Analysis

si-ASNR- or si-NC-transfected MKN45 cells and pcDNA3.1-ASNR- or blank vector–transfected MKN28 cells were obtained after 48 h of transfection by Lipofectamine 2000 (Invitrogen). Next, the cells were dyed with propidium iodide (PI) utilizing the DNA content quantitative measurement method (cell cycle) (Solarbio) and detected *via* FACScan. Calculate and compare the percentage of cells in G0/G1, S, and G2/M phases.

### Wound-Healing Assay

To conduct the wound healing experiment, 3 × 10^5^ cells were seeded into six-well plates and cultured overnight. Cells were transfected with pcDNA3.1-ASNR, si-ASNR, and si-NC, respectively. When the cultured cells reached a density of 90%, a sterile 10-ml disposable pipette tip was used to produce uniform scratches. The cells were then grown in a medium containing 1% FBS for 48 h. At indicated time points, a microscope was used to obtain images of the plate (Olympus, Tokyo, Japan). Meanwhile, scratch area and wound-healing percentage were calculated. The experiment was repeated three times in parallel.

### Cell Migration and Invasion Assays

si-ASNR- or si-NC transfected MKN45 cells and pcDNA3.1-ASNR-transfected MKN28 cells were harvested after 48 h of transfection and then collected. Twenty-four-well Transwell plates (8 μm pore size) (Corning Inc, Corning, NY, United States) were applied for cell migration and invasion assays. Briefly, 50 μl of Matrigel was placed into the Transwell upper chambers (Sigma-Aldrich). Cells (1 × 10^5^) suspended in 100 μl of serum-free culture medium were seeded into the upper chambers. Added RPMI 1640 culture medium supplemented with 10% FBS into the wells under the chamber. The cells were cultured in the medium for 48 h at 37°C with 5% CO_2_. Then, a cotton swab was utilized to wipe away the remaining cells on the membrane. Cells migrated or invaded through the membrane were stained with methanol and 0.1% crystal violet. Cells were then imaged and counted using an IX71 inverted microscope (Olympus). Five random fields were counted in each well. The experiment was repeated three times in parallel.

### Western Blot Assay and Antibodies

Radio immunoprecipitation assay (RIPA) protein lysis buffer (Solarbio) supplemented with protease inhibitor cocktail (Roche, Basel, Switzerland) and phenylmethylsulfonyl fluoride (Roche) was applied for protein extraction. The proteins concentration was evaluated through a BCA Protein Assay Kit (Solarbio). Ten percent sodium dodecyl sulfate–polyacrylamide gel electrophoresis (SDS-PAGE) was used to detach protein extracts (50 μg). The protein extracts were transferred to 0.22 mm polyvinylidene fluoride membranes (Sigma), then incubated with specific antibodies. ECL chromogenic substrate was applied to evaluate the specific bands. The specific bands were then quantified by densitometry (Quantity One software, Bio-Rad) with β-actin used as a control. All antibodies (1:2,000 dilutions) were purchased from Abcam (Cambridge, United Kingdom).

### Luciferase Reporter Assay

The 3′-UTR of human FGFR2 or Lnc_ASNR was amplified from human genomic DNA and individually inserted into a pGL3-basic vector (Promega, Madison, WI, United States). The fragment of FGFR2 or lncRNA-ASNR 3′-UTR mutant was then inserted into the pGL3-basic vector (Promega) control vector at the same position. To perform the reporter experiments, transfect MKN45 cells with wild-type or mutant reporter plasmid and miR-519e-5p mimic by Lipofectamine 2000 (Invitrogen). After 36 h of transfection, the luciferase activities and the firefly luciferase vitalities were detected under Dual-Luciferase^®^ Reporter Assay System (Promega). They were normalized based on Renilla luciferase activity.

### Immunohistochemistry

Formalin and paraffin were used to fix and embed the isolated tumor tissues, respectively. The tissue examples were then cut into 5 mm thick. Immunochemical staining was performed following the standard protocol. Tumors from mice were immunostained for H&E, Ki67. Inverted microscope (Nikon, Tokyo, Japan) was applied to capture the staining intensities of Ki67, and ImageJ software was used to analyze the data. Anti-Ki67 (1:50) was purchased from Roche.

### Xenograft Study

All experiments were approved by the Committee on the Ethics of Animal Experiments of Hebei Medical University. The investigations were conducted strictly on the basis of the recommendations in the *Guide for the Care and Use of Laboratory Animals of the National Institutes of Health*. MKN45 cells were stably infected with control shRNA or sh-ASNR using lentiviruses. GC cells were injected into either side of the armpit regions of the male BALB/c nude mice. The mice (4–5 weeks old) were randomly divided into two groups (*n* = 6/group). Tumor volumes and weights were measured every 3 days, and tumor volumes were measured regularly. Calculate as the equation: V = 0.5 × D × d^2^ (V, volume; D, longest diameter; d, diameter perpendicular to the longest diameter). After 16 days of treatment, mice were killed and tumors were excised. As mentioned earlier, H&E and IHC staining were performed on tumor tissues. The shRNA sequences used in our study were provided in the Supplementary Table 3.

### Statistical Analysis

All experimental data analyses were carried out using Student’s *t*-test and one-way ANOVA (GraphPad Prism 8). All data were presented as mean ± SD. The clinical correlations between two genes were analyzed by linear regression. The interrelation between the expression of ASNR and the clinical features in GC was confirmed by Fisher’s exact test. Impact of variables on survival was evaluated by univariate and multivariate Cox proportional hazard models. Overall survival (OS) rates were determined *via* Kaplan–Meier method. *P* < 0.05 was regarded as statistical significance.

## Results

### Lnc_ASNR Is Highly Expressed in GC and High Level of Lnc_ASNR Predicts Poor Prognosis

**FIGURE 1:**
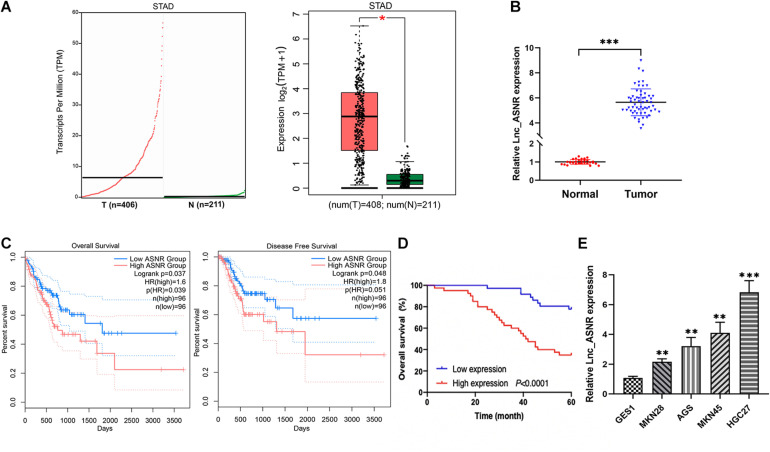
High expression of Lnc_ASNR in GC tissue samples and cell lines. **(A)** Lnc_ASNR expression was analyzed by using GEPIA2 in GC tissues and normal tissues. **(B)** qRT-PCR was applied to indicate the expression of Lnc_ASNR in GC tissue samples and adjacent non-tumor tissue samples (*n* = 76). **(C)** Overall survival and disease-free survival were analyzed using GEPIA2. **(D)** Kaplan–Meier analysis showed the relationship between Lnc_ASNR expression and overall survival time of GC patients. **(E)** qRT-PCR showed the expression of Lnc_ASNR in the normal cell line and GC cell lines. Error bars, mean ± SEM. *P < 0.05; **P < 0.01; ***P < 0.001.

Lnc_ASNR was highly expressed in the GC tissues by analyzing their expression level in the TCGA datasets and GTEx datasets through using GEPIA2 online web tool^1^ (Figure 1A). To determine Lnc_ASNR expression in GC tissues, real-time PCR was carried out in GC tissues and adjacent normal tissues. The result exhibited that Lnc_ASNR expression was obviously increased in GC tissue (Figure 1B). Those results together indicated that Lnc_ASNR was highly expressed in GC tissue. In addition, we also measured Lnc_ASNR expression in GES-1, MKN28, AGS, MKN45, and HGC27 cells. As shown in Figure 1E, Lnc_ASNR expression in the GC cell lines was obviously higher than that in the GES-1 cell. Compared with the highly differentiated cell line MKN28, the Lnc_ASNR expression was higher in the lowly differentiated cell line MKN45 (Figure 1E).

To evaluate the clinical significance of Lnc_ASNR overexpression in GC, we explored the expression of Lnc_ASNR in 76 patients with GC and evaluated their relationship with clinicopathological features. Median expression level was considered to be the key value. Tissues were grouped into low expression group with 36 samples and high expression group with 40 samples. As indicated in Table 1, high Lnc_ASNR levels was significantly associated with larger tumor size (*p* = 0.012), depth of invasion (*p* = 0.008), lymph node metastasis (*p* = 0.001), and TNM stage (*p* = 0.018). In contrast, no obvious association was found between Lnc_ASNR expression and other features including gender (*p* = 0.921) and age (*p* = 0.235). Correlation between Lnc_ASNR expression level and prognosis of GC patients was evaluated. According to the upper and lower quartiles of Lnc_ASNR expression, patients are divided into high-expression and low-expression groups. It was found that the expression of Lnc_ASNR affected the survival rate of the patient using GEPIA2 web tool. The overall survival (OS) and disease-free survival (DFS) of the low ASNR group were better than the high ASNR group (Figure 1C). It could be confirmed from Kaplan–Meier survival analysis that Lnc_ASNR highly expressed patients exhibited shorter OS, compared with Lnc_ASNR lowly expressed patients (Figure 1D). In addition, univariate survival analysis showed OS of patients were significantly correlated with that tumor size, infiltrating depth, Lauren classification, tissue differentiation, TNM stage, and Lnc_ASNR expression levels (Table 2). These factors were further evaluated by multifactor Cox regression analysis, indicating that Lnc_ASNR expression level and tissue differentiation are independent prognostic factors for patients (Table 3).

**TABLE 1:** Association of Lnc_ASNR expression with clinicopathological features of gastric cancer.

Features	Number	Lnc_ASNR expression levels		*X*^2^	*p*-Value
		High	Low		
**Gender**					
Male	46	24	22	0.010	0.921
Female	30	16	14		
**Age (years)**					
≤59	35	21	14	1.413	0.235
>59	41	19	22		
**Tumor size (cm)**					
≤4.8	48	20	28	6.283	0.012
>4.8	28	20	8		
**Lauren classification**					
Intestinal	21	7	14	11.689	0.003
Diffuse	30	23	7		
Mixed	25	10	15		
**Tissue differentiation**					
Well	5	0	5	12.110	0.001
Moderate	36	15	21		
Poor	35	25	10		
**Infiltrating depth**					
T1	3	0	3	10.717	0.008
T2	26	9	17		
T3	40	25	15		
T4	7	6	1		
**Nodal status**					
N0	18	3	15	16.223	0.001
N1	16	8	8		
N2	18	10	8		
N3	25	19	5		
**Distant metastasis**					
M0	74	38	36	1.849	0.495
M1	2	2	0		
**TNM stage**					
I	6	1	5	8.756	0.018
II	28	11	17		
III	40	26	14		
IV	2	2	0		

**TABLE 2:** Univariate analysis of prognostic factors in 76 patients with gastric cancer.

Characteristics	All cases	5-year survival rate	*X*^2^	*p*-Value
**Gender**				
Male	46	54.35% (25/46)	0.039	0.842
Female	30	56.67% (17/30)		
**Age (years)**				
<60	35	54.29% (19/35)	0.025	0.874
≥60	41	56.10% (23/41)		
**Tumor size (cm)**				
≤4.8	48	66.67% (32/48)	6.853	0.009
>4.8	28	35.71% (10/28)		
**Lauren classification**				
Intestinal	21	76.19% (16/21)	8.143	0.017
Diffuse	30	36.67% (11/30)		
Mixed	25	60.00% (15/25)		
**Tissue differentiation**				
Well	5	100.00% (5/5)	6.521	0.037
Moderate	36	61.11% (22/36)		
Poor	35	42.86% (15/35)		
**Infiltrating depth**				
T1	3	100.00% (3/3)	9.041	0.019
T2	26	69.23% (18/26)		
T3	40	50.00% (20/40)		
T4	7	14.29% (1/7)		
**Nodal status**				
N0	18	72.22% (13/18)	3.208	0.361
N1	16	50.00% (8/16)		
N2	18	44.44% (8/18)		
N3	25	52.00% (13/25)		
**Distant metastasis**				
M0	74	56.76% (42/74)	2.537	0.197
M1	2	0.00% (0/2)		
**TNM stage**				
I	6	83.33% (5/6)	7.253	0.041
II	28	67.86% (19/28)		
III	40	45.00% (18/40)		
IV	2	0.00% (0/2)		
**ASNR expression**				
Low	36	75.00% (27/36)	10.777	0.001
High	40	37.50% (15/40)		

**TABLE 3:** Multivariate analysis of prognostic factors in 76 patients with gastric cancer.

Clinicopathological features	*B*	*SE*	Wald	*p*-Value	95% CI
Tumor size (cm)	0.350	0.515	0.462	0.497	0.257∼1.934
Lauren classification	1.974	1.185	2.775	0.096	0.706∼73.446
Tissue differentiation	3.301	1.228	7.228	0.007	2.446∼300.984
Infiltrating depth	0.104	0.739	0.020	0.888	0.261∼4.724
TNM stage	1.789	1.246	2.062	0.151	0.520∼68.760
ASNR expression	2.772	0.821	11.400	0.001	0.013∼0.313

### Knockdown of Lnc_ASNR Inhibits While ASNR Overexpression Promotes GC Cell Proliferation, Migration, and Invasion

**FIGURE 2:**
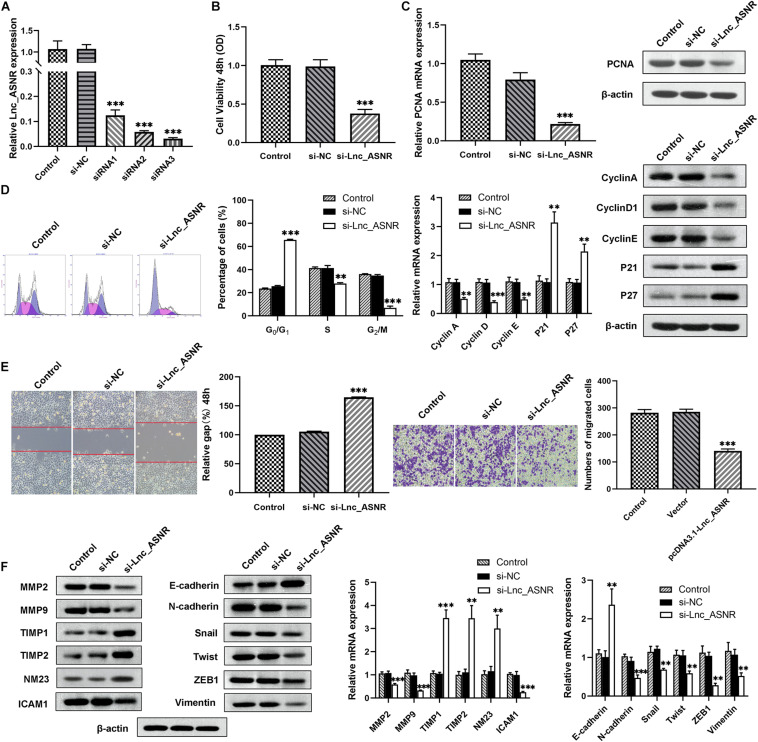
Silenced Lnc_ASNR inhibits GC cell proliferation, migration, and invasion. **(A)** qPCR showed the silence efficiency of Lnc_ASNR siRNAs. **(B)** MTT experiments detected the cell viability of MKN45 cells after treatment with Lnc_ASNR siRNA or control siRNAs. **(C)** qPCR and western blot assays showed PCNA mRNA and protein levels in si-Lnc_ASNR transfected MKN45 cells. **(D)** Flow cytometry was applied to verify cell cycle in MKN45 cell lines and qPCR and western blot assays were conducted to evaluate the cycle-related mRNA and protein levels. **(E)** The migration ability with Lnc_ASNR-silenced MKN45 cells was detected by wound-healing assay. The invasion ability was detected by invasion assay with Lnc_ASNR-silenced MKN45 cells. **(F)** The expression of metastasis-related mRNA and proteins with Lnc_ASNR-silenced MKN45 cells were investigated by performing qPCR and western blot assays. Error bars, mean ± SEM. *P < 0.05; **P < 0.01; ***P < 0.001.

We hypothesized that Lnc_ASNR may act as an oncogene, based on the increased expression of Lnc_ASNR in GC. Therefore, siRNA was used to downregulate the expression of Lnc_ASNR in MKN45 cells. qRT-PCR was used to verify knockdown efficiency of siRNA, and siRNA3 was chosen for subsequent experiments because of the highest knockdown efficiency (Figure 2A). Cell proliferation assay (MTT) results showed that silencing Lnc_ASNR inhibited the proliferation of GC cells (Figure 2B). It could be seen from the flow cytometry analysis that when Lnc_ASNR was silenced, the percentage of cells in G0/G1 phase would increase, while the percentage of cells in S phase would decrease (Figure 2D). The wound-healing assay and Transwell assay data suggested that compared with the control group, the si-Lnc_ASNR group significantly reduced the number of cells that migrated and invaded (Figure 2E).

To deeply verify whether Lnc_ASNR is related to GC, the expression of related mRNA and protein was evaluated by performing qRT-PCR and Western blot experiments. We found that both the mRNA and protein expression of PCNA, cell proliferation marker gene, was decreased (Figure 2C). Both mRNA and protein expression levels of cyclin A, cyclin D1, and cyclin E were decreased, while P21 and P27 expression levels were increased in the cells transfected with siRNA (Figure 2F). Furthermore, the mRNA and protein expression levels of MMP2, MMP9, and ICAM1, cell invasion- and migration-related gene, were decreased, while TIMP1, TIMP2, and NM23 were increased (Figure 2F).

**FIGURE 3:**
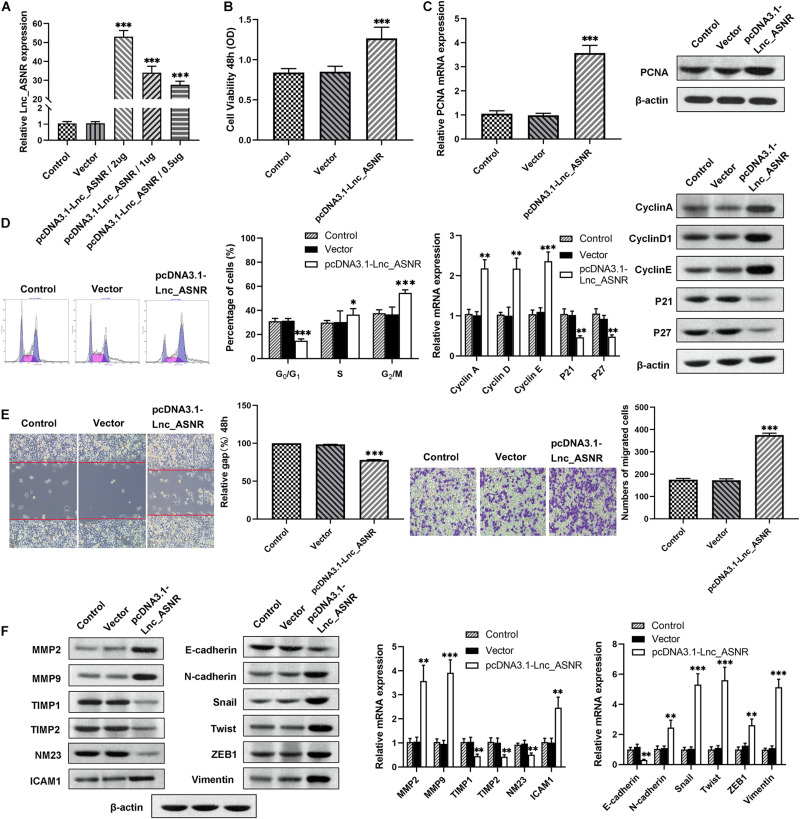
Lnc_ASNR overexpression promotes GC cell proliferation, migration, and invasion. **(A)** qPCR detected overexpress efficiency of pcDNA3.1-Lnc_ASNR. **(B)** MTT assays examined the cell viability of MKN28 cells after treatment with pcDNA3.1-Lnc_ASNR or vector. **(C)** PCNA mRNA and protein level in pcDNA3.1-Lnc_ASNR-transfected MKN28 cells were evaluated by carrying out qPCR and western blot assays. **(D)** Cell cycle was verified by using flow cytometry in MKN28 cell lines and cycle-related mRNA and protein level were evaluated by qPCR and western blot assays. **(E)** The migration ability with Lnc_ASNR-overexpressed MKN28 cells was detected by wound-healing assay. The invasion ability was detected by invasion assay with Lnc_ASNR-overexpressed MKN28 cells. **(F)** The expression of metastasis-related mRNA and proteins with Lnc_ASNR overexpressed MKN28 cells were investigated by performing qPCR and western blot assays. Error bars, mean ± SEM. *P < 0.05; **P < 0.01; ***P < 0.001.

Contrary to the results of silencing Lnc_ASNR, overexpression of Lnc_ASNR facilitated the proliferation, migration, and invasion of MKN28 cells (Figures 3A–F).

### Lnc_ASNR Influences GC Cell EMT

We found that cell migration and invasion are closely related to Lnc_ASNR levels. Since epithelial-mesenchymal transition (EMT) is a key step that contributes to tumor metastasis, we evaluated the effect of Lnc_ASNR on EMT. In order to further verify the abovementioned results, qRT-PCR and Western blot assay were conducted to evaluate the EMT-induced marker expression in cells downregulating or upregulating Lnc_ASNR. As shown in Figure 2F, high expressed epithelial markers E-cadherin, low expressed interstitial markers *N*-cadherin and vimentin were detected in the cells downregulated by Lnc_ASNR, indicating that the inhibition of Lnc_ASNR promoted the change of EMT to MET. Moreover, EMT transcription factor expression, including Snail, Twist, and ZEB1, was significantly downregulated (Figure 2F) in si-Lnc_ASNR-transfected cells. However, the results observed in the cells transfected with pcDNA3.1-Lnc_ASNR were just the opposite (Figure 3F). These results are consistent with our hypothesis that Lnc_ASNR affects the malignant phenotype by regulating EMT.

### Lnc_ASNR Promotes GC Cell Tumorigenesis *in vivo*

**FIGURE 4:**
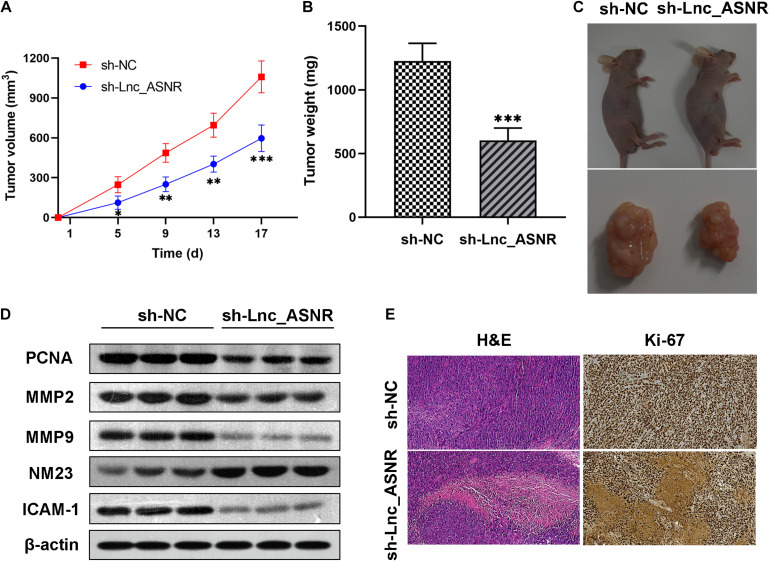
Silenced Lnc_ASNR suppresses the tumorigenesis ability *in vivo*. **(A)** The sizes of tumors were measured, and the volume was calculated every 4 days. **(B)** Tumor weights in different groups were shown. **(C)** Images of tumors formed from AGS cells in different groups were represented. **(D)** Related protein expression levels in tumor tissues were investigated *via* western blot assays. **(E)** Images of H&E staining and Ki67 immunostaining for nude mice tumor tissues in different groups. Error bars, mean ± SEM. *P < 0.05; **P < 0.01; ***P < 0.001.

In order to provide further evidence for the above findings, stably expressed sh-Lnc_ASNR or sh-NC AGS cells were constructed by infecting cells with shRNA vectors. Then, in order to determine the correlation between Lnc_ASNR and tumorigenesis of GC cells *in vivo*, AGS cells with stable knockdown of Lnc_ASNR were injected into nude mice. In contrary to the tumor formed by the control cells, the tumor formed by the Lnc_ASNR-silenced cells has taken significantly slower time (Figure 4A). In addition, compared with the control group, tumor volume of the sh-Lnc_ASNR group was significantly decreased (Figures 4B,C). We also found that the protein expression of cell proliferation marker gene, PCNA, and cell invasion and migration-related gene, MMP2, MMP9, and ICAM1, were decreased, while NM23 was increased in the sh-Lnc_ASNR group (Figure 4D). The above results demonstrated that the downregulated Lnc_ASNR could suppress tumor development *in vivo*. Furthermore, the results of H&E staining indicated that the sh-Lnc_ASNR group significantly suppressed tumorigenesis. Additionally, immunohistochemical staining of tumor tissues demonstrated that contrary to the sh-NC group, a decrease in Ki-67 was detected in the sh-Lnc_ASNR group (Figure 4E).

*In vivo* investigations above complement the evidence of *in vitro* functional studies involving Lnc_ASNR.

### Lnc_ASNR Functions as ceRNA and Sponges miR-519e-5p in GC Cells

**FIGURE 5:**
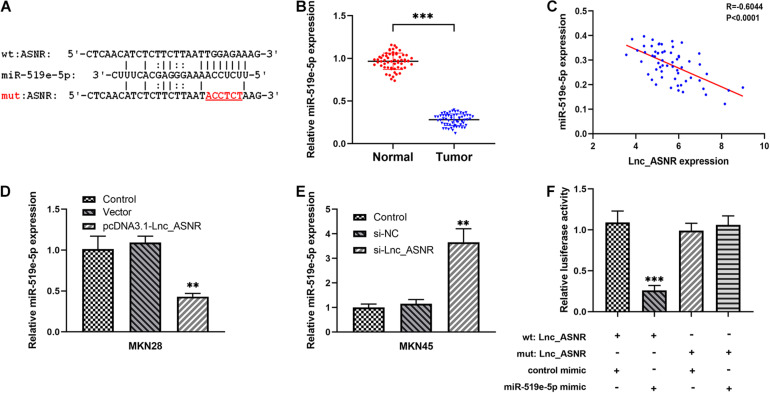
The relationship between Lnc_ASNR and miR-519e-5p. **(A)** The predicted combining positions between Lnc_ASNR and miR-519e-5p were predicted by using “miRDB” website (http://mirdb.org/). **(B)** qRT-PCR was applied to indicate the expression of miR-519e-5p in GC tissue samples and adjacent non-tumor tissue samples. **(C)** Correlation analysis was used to evaluate the relationship between miR-519e-5p expression and Lnc_ASNR expression. **(D)** qPCR detected the levels of miR-519e-5p in GC cells after Lnc_ASNR was overexpressed. **(E)** qPCR detected the levels of miR-519e-5p in GC cells after Lnc_ASNR was silenced. **(F)** Luciferase activity detection. Error bars, mean ± SEM. *P < 0.05; **P < 0.01; ***P < 0.001.

In addition to regulating the target in epigenetics, some lncRNAs can also act as competing endogenous RNA (ceRNA) of specific miRNA to affect the target gene expression. We predicted the potential target of Lnc_ASNR by searching the biological information database miRDB. We examined the Lnc_ASNR sequence and found the miR-519e-5p-binding site and identified miR-519e-5p as a candidate binding miRNA (Figure 5A). In order to detect the miR-519e-5p expression in GC tissues, real-time PCR was performed in GC tissues and matched adjacent normal tissues. It was clearly indicated that the expression of miR-519e-5p was markedly downregulated in GC samples (Figure 5B). Besides, Lnc_ASNR expression was negatively correlated with that of miR-519e-5p in GC samples (Figure 5C). We attempted to analyze the regulation of miR-519e-5p by Lnc_ASNR. Highly differentiated MKN28 cells were transfected pcDNA3.1(+) vector carrying the coding sequence of Lnc_ASNR to overexpress Lnc_ASNR, and downregulation of miR-519e-5p expression was detected by qPCR (Figure 5D). Whereas, lowly differentiated MKN45 cells were transfected with si-Lnc_ASNR to silence Lnc_ASNR and upregulated miR-519e-5p expression was detected by qPCR (Figure 5E). To further verify the targeting relationships between miR-519e-5p and Lnc_ASNR, luciferase reporter experiment was performed in MKN45 cells. As represented in Figure 5F, miR-519e-5p mimic apparently decreased the activity of luciferase in Lnc_ASNR-wild, while no significant change was detected in Lnc_ASNR-mut.

Therefore, these data suggested that Lnc_ASNR acts as a miR-519e-5p sponge in GC.

### FGFR2 Is a Downstream Target of miR-519e-5p

**FIGURE 6:**
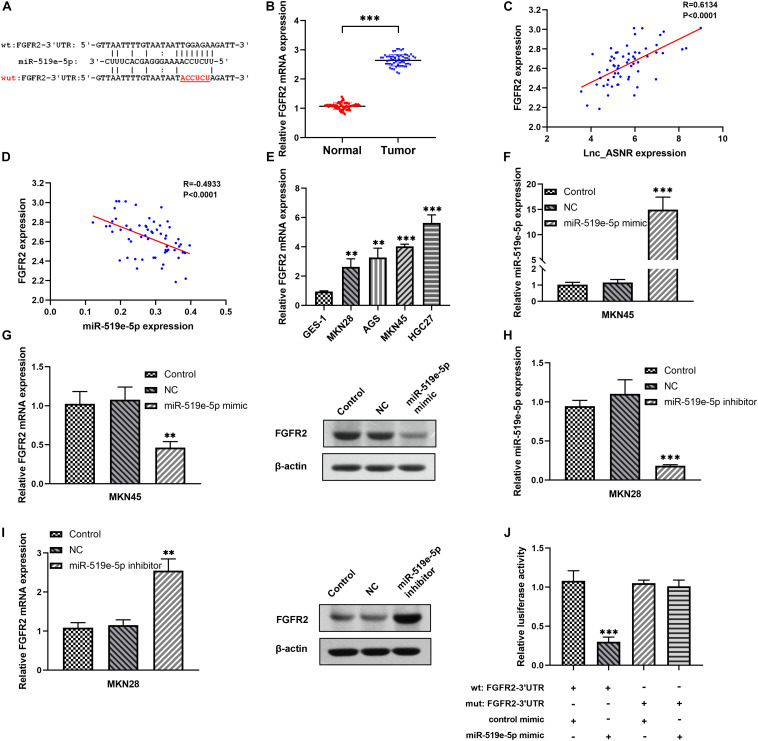
The relationship between FGFR2 and miR-519e-5p in GC cells. **(A)** The potential binding sites between miR-519e-5p and FGFR2 were predicted from “Targetscan” website (http://www.targetscan.org/) and “miRDB” website (http://mirdb.org/). **(B)** qRT-PCR was applied to indicate the expression of FGFR2 in GC tissue samples and adjacent non-tumor tissue samples. **(C)** Correlation analysis was used to evaluate the relationship between FGFR2 expression and Lnc_ASNR expression. **(D)** Correlation analysis was used to evaluate the relationship between FGFR2 expression and miR-519e-5p expression. **(E)** qRT-PCR showed the expression of FGFR2 in the normal cell line and GC cell lines. **(F)** qPCR detected the overexpress efficiency of miR-519e-5p mimic. **(G)** Relative FGFR2 expression and protein levels in GC cells after miR-519e-5p was overexpressed. **(H)** qPCR detected the silence efficiency of miR-519e-5p inhibitor. **(I)** Relative FGFR2 expression and protein levels in GC cells after miR-519e-5p was silence. **(J)** Luciferase activity examination. Error bars, mean ± SEM. *P < 0.05; **P < 0.01; ***P < 0.001.

Online miRNA target prediction databases (including Targetscan and miRDB) were applied to predict potential target genes of miR-519e-5p, and FGFR2 was determined as a potential downstream gene of miR-519e-5p (Figure 6A). Real-time PCR was carried out in GC tissues and matched adjacent normal tissues to evaluate the FGFR2 expression. An obvious upregulation of FGFR2 expression was detected in GC tissue samples (Figure 6B). Besides, FGFR2 expression was positively correlated with the expression of Lnc_ASNR (Figure 6C) and negatively related to miR-519e-5p expression in GC tissue samples (Figure 6D). In addition, FGFR2 expression in GES-1, MKN28, AGS, MKN45, and HGC27 cells was also detected. It was clearly indicated from Figure 6E that FGFR2 expression was markedly higher in GC cell lines than that of control cells.

Next, low differentiated MKN45 cells were transfected with miR-519e-5p mimic, and qRT-PCR was used to verify that miR-519e-5p expression was markedly increased (Figure 6F). In addition, the FGFR2 expression level was evaluated by qRT-PCR and Western blot experiments, and the data demonstrated that the FGFR2 expression level was markedly downregulated (Figure 6G). On the contrary, miR-519e-5p inhibitor was used to transfect highly differentiated MKN28 cells, and corresponding results demonstrated that the expression of miR-519e-5p was markedly decreased (Figure 6H). Moreover, FGFR2 expression was investigated by conducting qRT-PCR and Western blot experiments, and the results suggested that FGFR2 expression was obviously increased (Figure 6I). To further evaluate the targeting correlation between miR-519e-5p and FGFR2, luciferase reporter experiment was performed in the MKN45 cells. As exhibited in Figure 6J, miR-519e-5p mimic markedly suppressed the vitality of luciferase in FGFR2-wild, while no significant change was detected in FGFR2-mut.

Therefore, these data indicated that FGFR2 is an important downstream target of miR-519e-5p.

### The Lnc_ASNR/miR-519e-5p/FGFR2 Axis Promotes Proliferation, Invasion, and Migration of GC

**FIGURE 7:**
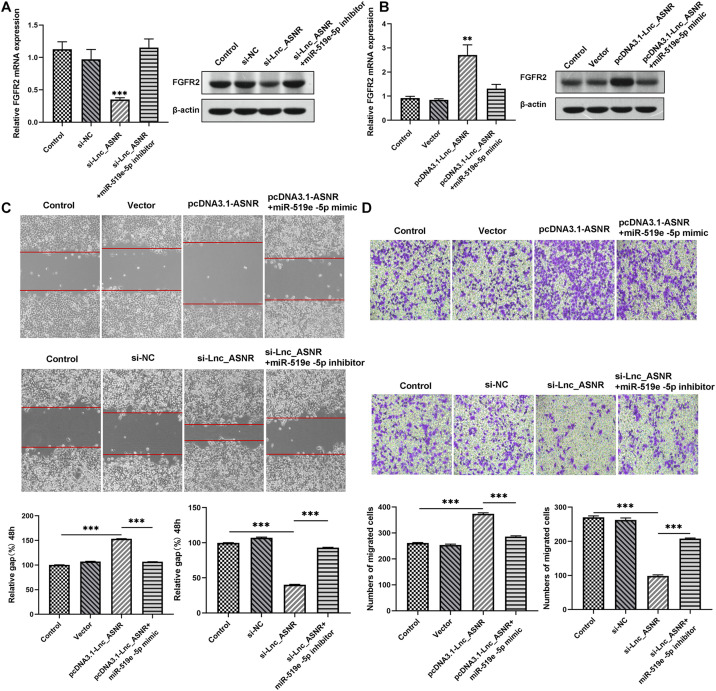
Lnc_ASNR/miR-519e-5p axis regulates GC progression. **(A)** The effects of Lnc_ASNR inhibition on the expression of FGFR2. **(B)** The effects of Lnc_ASNR overexpression on the expression of FGFR2. **(C)** The rescue experiments about si-Lnc_ASNR and pcDNA3.1-ASNR for cell migration. **(D)** The rescue experiments about si-Lnc_ASNR and pcDNA3.1-ASNR for cell invasion. Error bars, mean ± SEM. *P < 0.05; **P < 0.01; ***P < 0.001.

Mechanism research was conducted to clarify the relationship between Lnc_ASNR and FGFR2. Lnc_ASNR was silenced in lowly differentiated MKN45 cells. qRT-PCR and western blot assay inhibited that decreased Lnc_ASNR reduced the level of FGFR2 protein, which is consistent with the miR-519e-5p-induced FGFR2 protein downregulation. When transfected with si-ANSR + miR-519e-5p inhibitor in MKN45 cells, it was found that FGFR2 expression was restored (Figure 7A). In contrast, upregulated FGFR2 was detected in highly differentiated MKN28 cells who overexpressed Lnc_ASNR. When pcDNA3.1-ASNR + miR-519e-5p mimic was transfected in MKN45 cells, it was found that FGFR2 expression was reversed (Figure 7B). The effect of Lnc_ASNR/miR-519e-5p axis on GC was then evaluated. MKN28 cells were transfected with vectors, pcDNA3.1-ASNR, and pcDNA3.1-ASNR + miR-519e-5p minic, respectively. MKN45 cells were transfected with si-NC, si-ASNR, and si-ASNR + miR-519e-5p inhibitors, respectively. As shown in Figures 7C,D, miR-519e-5p mimics could counteract the enhancement effect of overexpression of Lnc_ASNR on cell migration and invasion ability, while miR-519e-5p inhibitor could reverse the inhibitory effect of knockdown of Lnc_ASNR on cell migration and invasion ability.

**FIGURE 8:**
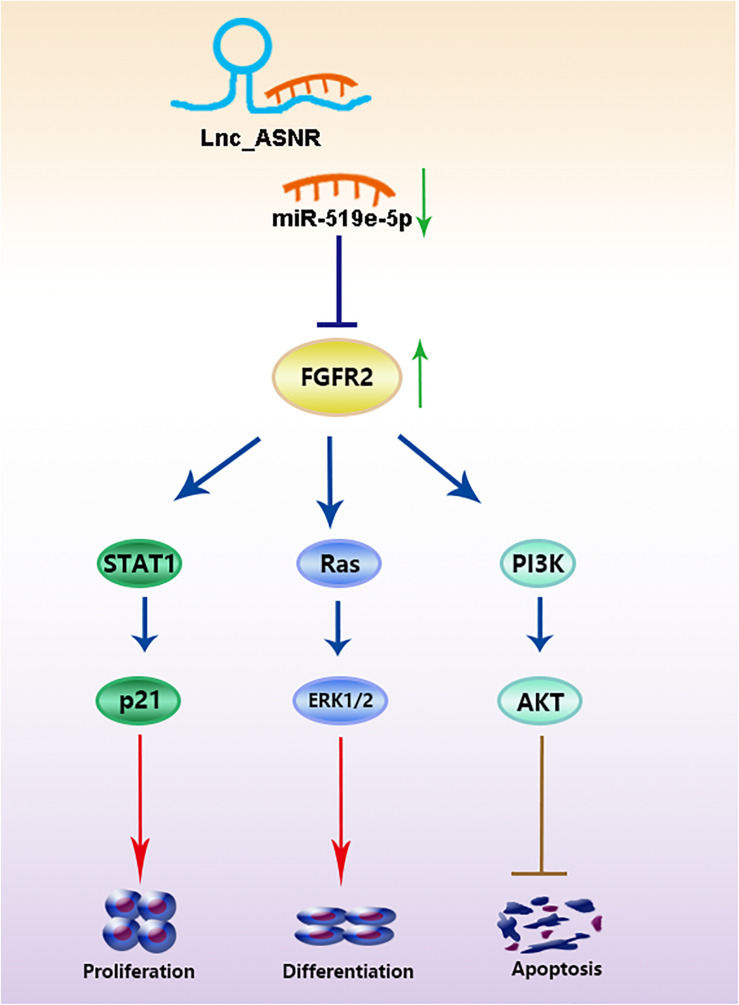
Lnc_ASNR acted as a ceRNA for miR-519e-5p promoting GC development by regulating FGFR2.

Ultimately, correlation analysis in GC showed that Lnc_ASNR expression and miR-519e-5p expression was negatively related, while Lnc_ASNR expression and FGFR2 expression was positively related. Overall, the above results demonstrated that Lnc_ASNR acted as a ceRNA for miR-519e-5p, thereby reducing FGFR2 expression and imposing posttranscriptional regulatory levels (Figure 8).

## Discussion

Generally, explorations for cancer drivers are focused on protein-coding genes in cancer genomes before the discovery of non-coding RNAs. However, increasing research results verified that lncRNAs are crucial in cellular progression and diseases development (Han et al., 2019; Pradas-Juni et al., 2020), especially in cancer (Papaioannou et al., 2019; Schulte et al., 2020; Silva-Fisher et al., 2020). Abnormal expression of lncRNAs may lead to accelerated tumor growth (Geng et al., 2018; Lin and Yang, 2018; Sallam et al., 2018). The therapeutic methods available to advanced GC patients are limited, and there remains potential to improve the treatment effect. It is essential to investigate thoroughly the internal mechanisms of GC occurrence and development and explore novel prognostic markers. Large amounts of lncRNAs are verified to play crucial roles in GC development. Increasing researchers pay more attention to investigate the functions and regulation of lncRNAs to explore novel predictors for the diagnosis and therapeutics of GC (Fu et al., 2020; Qin et al., 2020; Ren et al., 2020). Herein, a GC-associated lncRNA ASNR was identified, which was markedly increased in GC cells and tissues. High Lnc_ASNR expression was related to advanced TNM staging, larger tumor size, and positive lymph node metastasis. In addition, the upregulated expression of Lnc_ASNR was related to the shortening of OS and DFS in GC patients. The above investigations showed that knocking down Lnc_ASNR suppressed cell proliferation, declined tumor growth, and facilitated cell apoptosis, while upregulated Lnc_ASNR accelerated cell proliferation, invasion, and metastasis. These functional evidences indicate that Lnc_ASNR plays a carcinogenic role in the occurrence of GC, which can be used as a potential prognostic indicator.

Increasing evidences indicate that there is a novel and extensive interaction network involving ceRNA. It was reported that ncRNAs could regulate target RNA through combining their linking position of protein coding messengers (Wang P. et al., 2020; Wang W. et al., 2020; Zhao et al., 2020). Emerging researches confirm the ceRNA hypothesis and indicate that ceRNA regulation is related to the carcinogenic effects of GC. For example, LncRNA MT1JP can suppress cell growth, migration, and invasion, promote cell apoptosis *in vitro*, and inhibit tumor growth and metastasis *in vivo* (Zhang et al., 2018). In our previous investigations, we suggested that lncRNA PWRN1 acts as a ceRNA-targeting miR-425-5p and inhibits the growth of GC by intervening p53 signaling pathway (Chen et al., 2018). Thus, we assumed that lncRNA may play the role of ceRNA and participate in occurrence of GC. It was confirmed by bioinformatics analysis and luciferase reporter gene analysis that miR-519e-5p was targeted by Lnc_ASNR. Recently, miR-519e-5p has only been reported in acute myocardial infarction (Wang F. et al., 2014) and pregnancy-related complications (Hromadnikova et al., 2015; Zhang et al., 2016), while few investigations on the relationship between miR-519e-5p and GC were reported. In our research, we confirmed that the endogenous level of Lnc_ASNR could influence the miR-519e-5p expression. The miR-519e-5p expression was increased in GC cells when Lnc_ASNR was silenced. Contrary to the results of silencing Lnc_ASNR, miR-519e-5p expression was decreased upon Lnc_ASNR overexpression, which confirmed our hypothesis. Above investigations revealed the importance of the correlation between Lnc_ASNR with miR-519e-5p in GC, which was contributed to that Lnc_ASNR exhibited a carcinogenic effect through causing miR-519e-5p to produce sponges in GC cells.

In general, lncRNAs exhibit function by relying on ceRNA to inhibit miRNA targets (Shen et al., 2018; Chen et al., 2019; Yang et al., 2019). Therefore, miRNA targets are important parts of ceRNA network. It is worth to note that FGFR2 is a potential miR-519e-5p target site which has never been reported *via* online-predicting database. In order to verify that miR-519e-5p was directly targeted by FGFR2, we carried out luciferase reporter experiments and confirmed that FGFR2 was targeted by miR-519e-5p at 3′-UTR. In addition, transfection of miR-519e-5p mimic inhibits FGFR2 mRNA and protein expression, while transfection of miR-519e-5p inhibitor promotes FGFR2 mRNA and protein expression. The protein encoded by the FGFR2 gene is one of the members of the fibroblast growth factor receptor (FGFR) family (Wu et al., 2013). At present, four types of FGFRs have been identified, namely FGFR1, FGFR2, FGFR3, and FGFR4. The main function of FGFR, a receptor for FGF, is to transduce FGF signal to RAS-ERK and PI3K-AKT signal cascade amplification. The missense mutations of the FGFR2 gene occur in endometrial cancer, cervical cancer, breast cancer, lung cancer, and GC. The amplified FGFR2 induces the activation of FGFR2 signaling. FGFR2 has been proposed as a target for targeted therapy of GC (Pearson et al., 2016; Kuboki et al., 2018; Kim et al., 2019). Yu et al. (2018) revealed that overexpressed FGFR2 accelerated the production of cancer-initiating cells (CIC) and enhanced the resistance to lapatinib in HER2-positive GC cells. We further determined that miR-519e-5p could reverse the effect of Lnc_ASNR on FGFR2 expression level, indicating that Lnc_ANSR acts on miR-519e-5p through sponge adsorption, which further affects the expression of downstream target gene FGFR2, thereby affecting cell proliferation, invasion, and migration activity.

In summary, our findings validated a novel lncRNA, called Lnc_ASNR, and found that Lnc_ASNR expression was related to poor prognosis in GC. The study found that Lnc_ASNR was a carcinogenic lncRNA exhibiting a targeting relationship with miR-519e-5p. GC patients with higher expression of Lnc_ASNR promoted tumor growth by regulating the miR-519e-5p/FGFR2 pathway. The current study reveals a further insight into the function of lncRNA–miRNA–mRNA–ceRNA network in GC progression. The promoter function of Lnc_ASNR in the GC progress was first reported. The potential molecular mechanism involved in the Lnc_ASNR/miR-519e-5p/FGFR2 axis could be a potentially useful approach for GC diagnosis and therapies.

## Data Availability Statement

The original contributions presented in the study are included in the article/Supplementary Material, further inquiries can be directed to the corresponding author/s.

## Ethics Statement

The animal study was reviewed and approved by Research Ethics Committee at the Fourth Hospital of Hebei Medical University. Written informed consent was obtained from the individual(s) for the publication of any potentially identifiable images or data included in this article.

## Author Contributions

ZC and YLo planned, designed, and coordinated this research and drafted the manuscript. BT and QZ participated in the conception and design of the manuscript and revised it critically for important intellectual content. FL and LF contributed to the conception and design and helped in drafting the manuscript. ZZ, XZ, and YLu contributed to analyzing and interpreting data and improved the crucial parts. DW helped in writing a part of the manuscript and analyzed the data. All the authors read and approved the final manuscript.

## Conflict of Interest

The authors declare that the research was conducted in the absence of any commercial or financial relationships that could be construed as a potential conflict of interest.

## Footnotes

1 http://gepia2.cancer-pku.cn/

## Abbreviations

GC: gastric cancer
lncRNAs: long non-coding RNAs
qRT-PCR: quantitative real-time PCR
MTT: 3-(4,5)-dimethylthiahiazo (-z-y1)-3,5-di-phenytetrazoliumromide
EMT: epithelial-mesenchymal transition
FGFR: fibroblast growth factor receptor
nt: nucleotides
ceRNA: competitive endogenous RNA
TNM: tumor-node-metastasis
si-NC: negative control siRNA
GFP: green fluorescent protein
RIPA: radio immunoprecipitation assay
OS: overall survival
CIC: cancer-initiating cells.
