# Supplementary material for: Long Non-coding RNA ASNR Targeting miR-519e-5p Promotes Gastric Cancer Development by Regulating FGFR2
Source: Front Cell Dev Biol. 2021 Jul 9;9:679176. doi: 10.3389/fcell.2021.679176 (PMC8299726; doi:10.3389/fcell.2021.679176)
Supplement: Supplementary file 1 [file Data_Sheet_1.docx]

**Table S1** The list of primers

| Gene | Forward primer | Reverse primer |
| --- | --- | --- |
| Lnc_ASNR | GAGAATTCCTCTGGTGGCTAAA | GGAACTGTAATCTCACTGGACAA |
| GAPDH | AATCCCATCACCATCTTCCAG | CCTTCTCCATGGTGGTGAAGAC |
| β-actin | TACCTCATGAAGATCCTCACCGA | AAGCATTTGCGGTGGACGAT |
| cyclin A | ATGTTGGGCAACTCGCGCCG | TTTCATCTTCTAATACAATTG |
| cyclin D1 | CAAGGCCTGAACCTGAGGAG | CTTGGGGTCCATGTTCTGCT |
| cyclin E | ATGCCGAGGGAGCGCAGGGAG | GGTCTCCTATGAAGTTTATAGAC |
| P21 | TGCAACTACTACAGAAACTGCTG | CAAAGTGGTCGGTAGCCACA |
| P27 | CCATGCCTGTAGTGATTA | CCCGACCCAGTTTGTCCA |
| MMP2 | CCAACTACAACTTCTTCCCTCG | TCACATCGCTCCAGACTTG |
| MMP9 | ACGCAGACATCGTCATCCA | AGGGACCACAACTCGTCATC |
| TIMP1 | TTCTGGCATCCTGTTGTTG | GTGGTCTGGTTGACTTCTGG |
| TIMP2 | CGACATTTATGGCAACCCT | ATTCCTTCTTTCCTCCAACG |
| NM23 | AAGGAGATCGGCTTGTGGTTT | CTGAGCACAGCTCGTGTAATC |
| ICAM1 | GTCATCATCACTGTGGTAGCAG | GGCTTGTGTGTTCGGTTTC |
| E-cadherin | GTGGTCAAAGAGCCCTTACTG | CGTTACGAGTCACTTCAGGC |
| N-cadherin | TCATTGCCATCCTGCTCTG | CATCCATACCACAAACATCAGC |
| Vimentin | AAATGGCTCGTCACCTTCG | AGAAATCCTGCTCTCCTCGC |
| Snail | TCGGAAGCCTAACTACAGCG | CAGAGTCCCAGATGAGCATTG |
| Twist | GTCCGCAGTCTTACGAGGAG | GCTTGAGGGTCTGAATCTTGCT |
| ZEB1 | GCTTCTCACACTCTGGGTCTTA | CCTCATTCTCTGCCTCTTCTACC |
| miR-519e-5p | CACATTGCTTACACCATTAGGC | TTCATTCTGATTTAGTAGGCTG |
| FGFR2 | TGGAGCGATCGCCTCACCG | CTTCCAGGCGCTGGCAGAACTGT |

**Table S2** Sequences for siRNA.

| siRNA | Sense (5'-3') |
| --- | --- |
| siRNA#1 | GGAGAAUGCAGAAUGGUCAGACUAC |
| siRNA#2 | GCAGGAACUUGAUUACUUUGAGUGC |
| siRNA#3 | ACACCAUUAGGCAUAGAUUCAGUGT |

**Table S3** Sequences for shRNA.

| shRNA | Sequence |
| --- | --- |
| shRNA#1 | AGTTCCATCTCACAGAAATTACTCGAGTAATTTCTGTGAGATGGAACT |
| shRNA#2 | TACACCATTAGGCATAGATTCCTCGAGGAATCTATGCCTAATGGTGTA |
| shRNA#3 | GTAATCTCACTGGACAATTAACTCGAGTTAATTGTCCAGTGAGATTAC |
